# Low-Cost Algorithms for Metabolic Pathway Pairwise Comparison

**DOI:** 10.3390/biomimetics7010027

**Published:** 2022-02-21

**Authors:** Esteban Arias-Méndez, Diego Barquero-Morera, Francisco J. Torres-Rojas

**Affiliations:** 1 Escuela de Computación, Instituto Tecnológico de Costa Rica, Cartago 30101, Costa Rica; ftorres@tec.ac.cr; 2 PaRMa Group, Instituto Tecnológico de Costa Rica, Cartago 30101, Costa Rica; 3 Ingeniería en Biotecnología, Instituto Tecnológico de Costa Rica, Cartago 30101, Costa Rica

**Keywords:** graph comparison, metabolic pathways, graph alignment, graph depth-first traversal, graph breadth-first traversal, global alignment, local alignment, semi-global alignment

## Abstract

Metabolic pathways provide key information for achieving a better understanding of life and all its processes; this is useful information for the improvement of medicine, agronomy, pharmacy, and other similar areas. The main analysis tool used to study these pathways is based on pathway comparison, using graph data structures. Metabolic pathway comparison has been defined as a computationally complex task. In a previous work, two new algorithms were introduced to treat the problem of metabolic pathway pairwise comparison. Here we provide an extended analysis with more data and a deeper analysis of metabolic pathway comparison as listed in the discussion and results section.

## 1. Introduction

In the last 50 years, at least, tools for data comparison on bioinformatics have been proposed, such as the Needleman–Wunsch algorithm in 1970 [1,2,3,4] as: *“A general method applicable to the search for similarities in the amino acid sequence of two proteins"*. This alignment algorithm has been key to arranging sequences of DNA, RNA, or proteins to identify regions of similarity that may be related to functional, structural, or evolutionary relationships between the entry sequences. Software tools like BLAST (first appeared in 1990) [5], which are widely used to this day, implemented, extended, and improved some of these algorithms.

There has been much work conducted on the analysis of network-based structures. In 1988, Bruce L. Clarke presented its stoichiometric network analysis, developed to study instability in inorganic chemical reaction networks, which relied on mass-action theory and used concepts of convex analysis [6]. Alex Seressiotis with James E. Bailey developed an artificial intelligence algorithm to search through reaction networks to identify and synthesize biochemical pathways [7]. Later on, in 1993, R. Schuster and S. Schuster introduced convex analysis first applied to metabolic networks [8]; these last two as examples of network-based structures.

## 2. Metabolic Pathways and Graphs

An ordered sequence of biochemical reacting metabolites, or a metabolic pathway, are chemical compounds that act as substrates to be transformed into other compounds (in this case, products) through a path of chemical reactions catalyzed by enzymes [9,10].

KEGG (www.genome.jp/kegg/) [11] (accessed on 1 March 2020) and MetaCyc (part of BioCyc https://biocyc.org) [12,13] (accessed on 1 March 2020) are examples of the best and most important databases of such data for several organisms from all kinds of taxa.

A work by [14] used computational methods to combine data from compounds and between different organisms, from databases such as BRENDA, ENZYME, and KEGG, allowing them to obtain the metabolic pathway of the Glycolysis process, essential to organisms to get energy (in the form of pyruvate, an energy-rich intermediary) from sugars (mainly glucose).

*Pairwise comparisons* of metabolic pathways for agronomic, pharmaceutical, medical, and commercial importance, also known as *pathway alignment*, *graph alignment*, or simply *alignment*, are current areas of research interest in metabolomics. Most of these comparisons can be represented as problems in the class of NP-Complete, which are very complex to solve, even for computers, as demonstrated in [1,2].

A better understanding of phylogenetic evolution, speciation and reconstruction [15,16] and the discovery of more effective drugs [17] may be possible thanks to the comparative analysis of different organism pathways.

Prior tools are based on the comparison using graph data structures. Different authors have proposed alternative ways, such as Ay and Kahveci [1] who proposed SubMAP, which focuses on finding common sub-parts between different pathways. The CAMPways algorithm from Abaka et al. [2] promises to be efficient at run-time.

Then, [18,19] applied heuristics to reduce the time taken by the algorithms of graph alignment, causing a loss of generality but making the data process more straightforward.

In this work, we will be using simple but important definitions necessary to clarify:

- *Node label*: for any given node in a graph, we refer to “label” as the associated string used to identify each node (which corresponds to the compound or metabolite name of a metabolic pathway represented by said node). Each label is unique within a pathway, meaning that two nodes in the same graph cannot have the same label name.
- *Equivalent nodes*: any given labeled node present in both graphs being compared, meaning that both pathways involve the use of the same compound described by the associated nodes.
- *Analogous order*: for two aligned sequences S and T, the elements with analogous order between both sequences are those that conform to the largest possible sub-sequence of both S and T.

It is not the intention of this work to give a definitive answer to the result of the metabolic pathway pairwise comparison problem or to indicate that one pathway is better than another one.

## 3. Algorithms

Two different algorithms as low-cost mechanisms for the pairwise comparison of metabolic-pathways as a previous step to a deeper analysis were proposed in [3]; below is a quick review.

### 3.1. Algorithm 1: Transformation of the 2D Pathway Graph to a 1D or Linear Structure for Later Alignment and Evaluation

**Figure 1:**
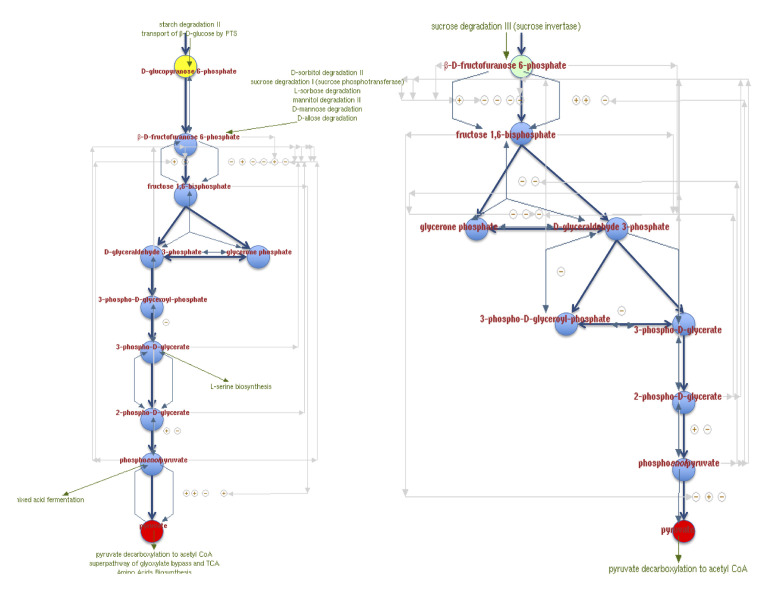
Glycolysis I and Glycolysis IV model of metabolic pathways as graphs.

**Figure 2:**
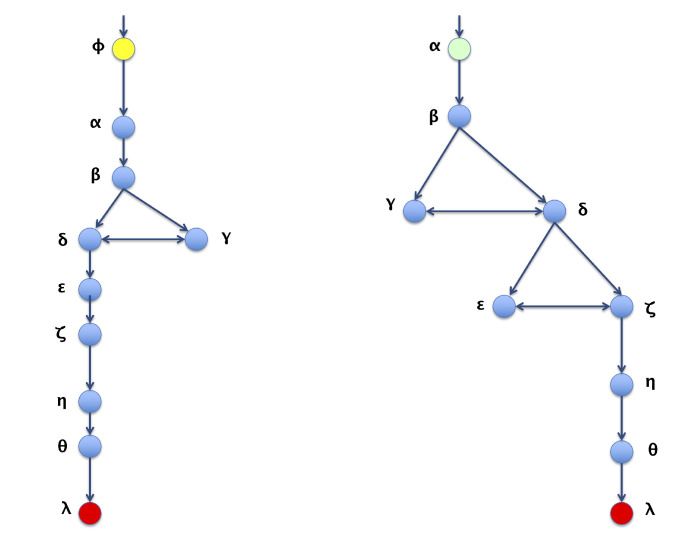
Nodes are relabeled according to their corresponding metabolites to simplify processing.

The figures below have metabolic pathways with common elements, equivalent nodes and homologous reactions, but quantitatively measuring their similarity is of interest. The first step for this analysis is to visualize both pathways as graphs (Figure 1) and label the nodes by their corresponding metabolites (Figure 2). It means that all the nodes of the graphs are distinguished. Equivalent nodes or equivalent reactions mean that the same metabolite or the same reacting metabolites are present in both graphs.

**Figure 3:**
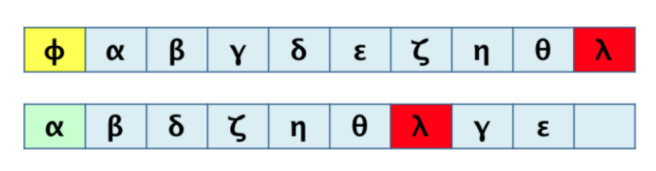
Depth-first traversals of pathways for graphs in Figure 2.

**Figure 4:**
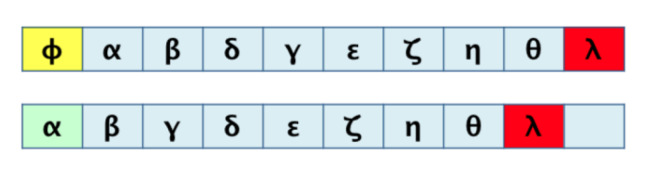
Breadth-first traversals of pathways for graphs in Figure 2.

For graphs that represent a metabolic pathway, a traversal lecture (which visits all the nodes) is helpful to get the series of elements using a selected root and, sometimes, the desired end; this is the required transformation from 2D to 1D. Common graph-traversal algorithms are depth-first [20] and breadth-first [21]; there is more about this on [22,23,24]. It has been observed that when applying a depth-first algorithm the information obtained is not relatively proportional and relevant to the route because the product may appear in the middle of the 1D row and not at the end of said row (as one might expect in a series of reactions which hold said product at the end). For example, depth-first traversal would result, as shown in Figure 3. When performing a breadth-first traversal, the nodes are visited by levels, which correspond more closely to how the metabolites’ reactions occur until the expected product is reached. Breadth-first traversal for the routes is shown in Figure 4.

**Figure 5:**
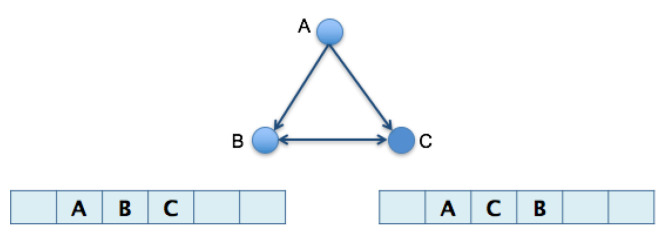
Possible loss of information due to 2D to 1D transformation.

Useful data correspond mainly to that generated by the breadth-first traversal algorithm. It should be noted that there will be a loss of information in such a transformation. Figure 5 shows this fact, mainly on the order of the elements and their original relationships. We look to demonstrate that such a loss of information during the process is tolerable and acceptable for a correct pairwise comparison result.

Once the pathway data are raised to obtain the traversal in a 1D format, we proceed to apply traditional sequence alignment techniques: global (GA) [4], local (LA) [25], and semi-global (SGA). We get a numeric values comparison of the sequences from the graphs.

**Figure 6:**
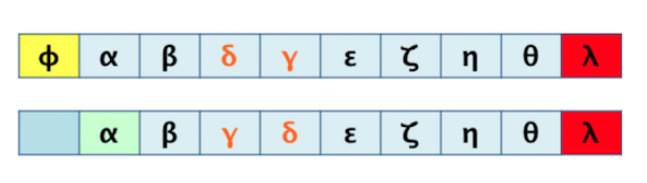
Global alignment generated for transformed graphs, the optimal value reached: +3.

**Figure 7:**
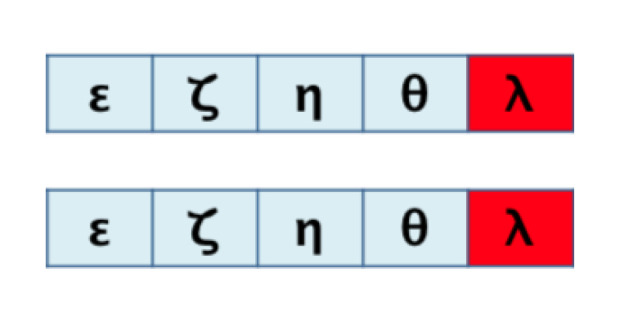
Local alignment generated for transformed graphs, the optimal value reached: +5.

A sample of the results of this process is summarized in Figure 6 and Figure 7.

### 3.2. Algorithm 2: Differentiation by Pairs

**Figure 8:**
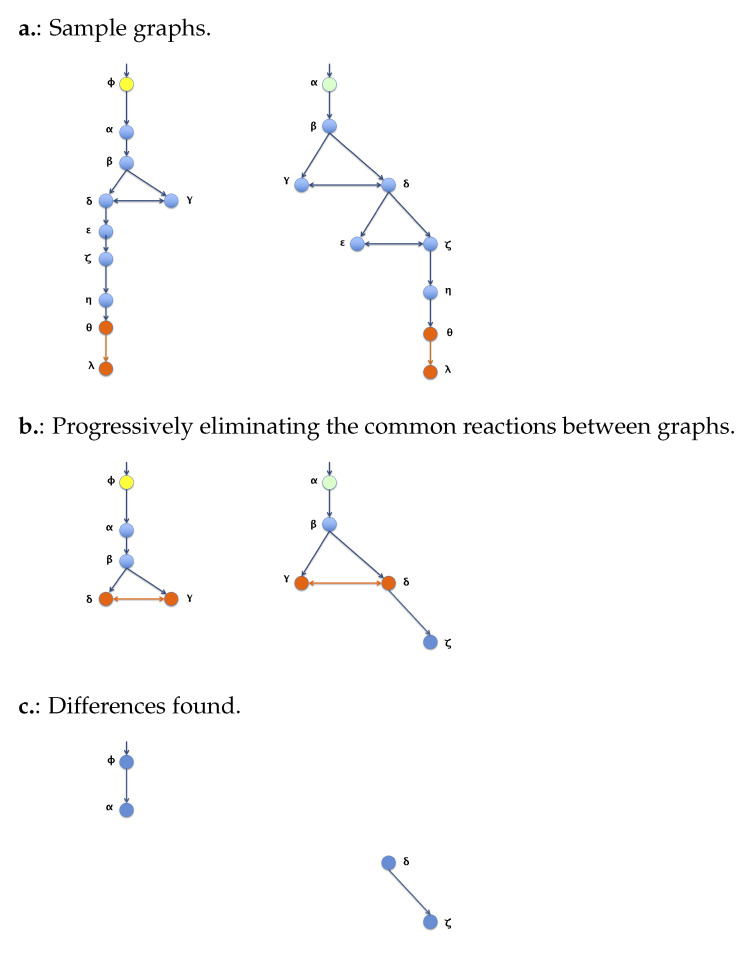
Peer differentiation process. (**a**) shows the original sample graphs been compared; (**b**) shows one of the intermediary steps; (**c**) shows the resulting differences found.

This method differs from a traditional numeric alignment of paths. The coincidences take a more relevant role than the differences between a given couple of routes. We also calculate a numeric value, using a relation between the number of differences and the total number of reactions, called “Numerical Differentiation by Pairs”. So far, no similar approach has been found, so the resulting data are more an intuitive homology for the user than a value of similarity. The results are later correlated to the numerical values of the first proposal to validate the differences found. The process is thoroughly explained in [3]. However, in Figure 8, a summary of the process is shown.

## 4. Materials and Methods

Here, we look for a proper way to validate the proposed algorithms and, for that, several tests have been completed, as we will review in the Results section. Besides other testings, we have also chosen a Design of Experiments (DoE) model, a formal test. The main reason for this is a great acceptance and extended use of it in several scientific projects [26].

Design of Experiments (DoE) is a systematic and rigorous approach to analyzing and resolving different problems [27]. It applies several principles and techniques to data collection to ensure a valid conclusion. We look to see if any factors may affect the results of the proposed algorithms in this work.

### 4.1. Factors and Levels

On DoE, a *factor* is a component that may influence the response variable [27]. The main objective of an experiment is to determine its influence. Each factor can have different possible *levels* for the experiment.

In the particular case of this work, we are considering metabolic pathways as the subject of study and a score of the pairwise comparison of them as the desired result. We look to make comparisons as unrestricted as possible, to be open to a higher possible amount of comparisons. Furthermore, we considered only two main characteristics that can be part of any given metabolic pathway: its size and its families.

Taking the metabolic pathways as directed graphs, we then considered mainly the sizes of each graph to be compared as a possible factor and specifically the size ratio between the pair graphs as the first factor. The second factor considered is the number of common families between the two graphs.

As explained before, the size ratio between graphs consists of dividing the smaller graph’s size (graph with the least amount of nodes) over the bigger graph’s size (graph with the largest number of nodes) to obtain a value between 0 and 1 that represents how different the sizes of the compared graphs are. This value can also be interpreted as how much of the biggest graph’s size can be covered by the smallest graph’s size. The higher the value, the more similar the sizes of both graphs.

There are many possible sizes and several ranges of common families. To simplify the comparison of the data, we group the values of the factors in three different ranges for each one. So, the selected criteria for this analysis are as follows:

1. Size ratio
  (a) much difference: x < 0.4, called different
  (b) mean difference: 0.4 ≤ x < 0.7, called medium
  (c) little difference: x ≥ 0.7, called similar
2. Common families
  (a) none common families: 0, called none
  (b) few common families: 1, 2, 3, called few
  (c) several common families: ≥4, called several

Table 1 summarizes this:

For each proposed algorithm, the desired result in both cases is a score, meaning a value of similarity between the pairwise compared metabolic pathways. As we will review in the next chapter, the provided scores are values between 0.0 and 1.0 as a percentage (%) of similitude. So, the response variable for both algorithms and their related analysis would be the resulting score obtained from each comparison executed.

### 4.2. Analysis of Variance: ANOVA

The ANOVA test (or Analysis of Variance) compares the mean of multiple groups. The term ANOVA is a little misleading. Although the technique’s name refers to variances, the main goal of ANOVA is to investigate differences in means. It makes it possible to ensure that the variation in the results of an experiment is not greater than the sum of the variations of the factors and a certain degree of error in their measurements. Statistical evidence allows the acceptance or rejection of the hypothesis with a probability of error (preferably very low) [26].

Two-way ANOVA was used to simultaneously evaluate the effect of two different grouping variables on a continuous outcome or response variable. Other synonyms are two factorial design, factorial ANOVA, or two-way between-subjects ANOVA. The independent grouping variables are also known as between-subjects factors. The main goal of two-way and three-way ANOVA is, respectively, to evaluate if there is a statistically significant interaction effect between two and three between-subjects factors in explaining a continuous outcome variable [28].

### 4.3. Obtaining the Data

We first considered two options, KEGG and MetaCyc; both are self-announced as public repositories. However, KEGG now has the KEGG FTP Academic Subscription available as a paid service by Pathway Solutions for academic users who wish to bulk download. We did not consider paying for this service as an option to obtain all the available data for testing purposes. Then, the database chosen for obtaining the pathways dataset was MetaCyc.

The MetaCyc database also focuses on individualizing the pathways into biologically meaningful units (occasionally distinguishable for individual organisms), instead of combining reaction and pathways from multiple species into a single chimeric pathway, such as KEGG. Nevertheless, MetaCyc does have super-pathways, which comprise multiple sub-pathways, but they usually do occur in a single organism, as explained in the MetaCyc User Guide (https://metacyc.org/MetaCycUserGuide.shtml) (accessed on 1 March 2020). The characteristic individualization of these pathways allows for a good random sample pool of graphs, as expected to be all kinds of comparisons, between contrasting or similar pathways. In contrast, the chimeric nature of data from other sources implies the unification of the would-be-compared graphs into single large units, making the process of randomly sampling for comparisons harder.

MetaCyc categorizes data in several ways, one of which is the organism to which the data belong. It does so by assigning an ID to each organism. The organism ID “META” is selected to obtain the pathways dataset, that is, the multi-organism pathway database containing general metabolic data and is not restricted to a single organism, as explained in the MetaCyc website (https://metacyc.org/PToolsWebsiteHowto.shtml#dbselect) (accessed on 1 March 2020). The database categorizes metabolic pathways according to their biological functions and metabolites involved with a hierarchy of pathway classes. Each class is composed of a large group of sub-classes and pathways, first grouping them by general characteristics (for example, “Biosynthesis”), and further down the hierarchy, forming detailed classes of pathways (for example, “*Glycogen and Starch Biosynthesis*”) (https://metacyc.org/META/new-image?object=Pathways) (accessed on 1 March 2020). We refer to those categories as “families” of metabolic pathways.

The websites with the hierarchy information for the organism ID “META” were automatically traversed, storing the ID of each pathway when found and the most detailed subclass (“family”) containing it. The XML file of each pathway is requested of the database, using the ID of each pathway. Then, the data are refined to store the described nodes and edges in JSON files in ReactionLayout (RNL) and DictionaryPathWay (DPW) formats. Basic pathways (those with no sub-pathways referenced in their XML file) are prioritized. Afterwards, super pathways are assembled, using the instructions for nodes and edges contained within the respective XML file and the already processed instructions for the reference sub-pathways (stored in DPW files). This way, a dataset of 3241 basic metabolic pathways and super pathways was obtained.

DictionaryPathWay or DPW format, a JSON file, consists of a directed graph data structure based on a dictionary where the keys are strings with the label of a node. The values are listed with the labels of the nodes directed. As a dictionary, this structure does not allow duplicate nodes and will merge the edges of duplicate nodes into a single key (merging also the associated lists). It is allowable for a metabolic pathway into a single chemical background. The nodes represent chemical compounds dispersed in a theoretically ubiquitous manner across the system, as long as no compartmentalization of the reactions is involved. It is the case that metabolic databases tend to miss compartmentalization when representing metabolic pathways [29].

ReactionLayout or RNL format, a JSON file, is another way of storing a directed graph data structure based on a dictionary. This time, the keys are strings with the database identifier for the reactions. The values are list data-structures that contain two internal lists: the first one stores the nodes’ label for the substrates of the reaction, while the second one stores the labels for the products.

### 4.4. Selection of Matching Candidates for the Comparisons

After gathering the metabolic pathway dataset, an automatic selection was conducted for the experimental comparisons. Meaningful scenarios, close to the actual practical use of the tool, were desired, so the selection process consisted of two criteria. The first indicates that both graphs must contain “origin” and “destiny” nodes to compare two pathways. An origin is a node that directs to one or more nodes, but none other node directs to it (i.e., analogous to the root of a tree data structure). While a destiny node is a node such that at least another node directs to it. However, it does not direct to any other node (i.e., a leaf on a tree data structure). Under this simplified biological context, the origin nodes would represent initial substrates for the pathway, while the destiny nodes would be the expected products of interest.

**Figure 9:**
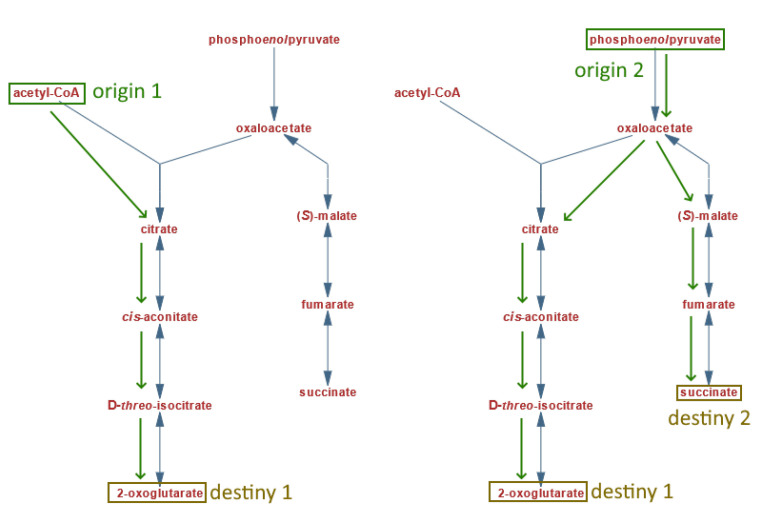
Possible traversals or lectures from a single pathway. Original figure from MetaCyc.

The second criterion for the selection process aims to detect which graphs from each pathway can generate valid lectures or traversals between the given origin and destiny nodes. It considers that it is possible to start the traversal of the graph from a given origin node but never reach the desired destiny node without starting another traversal from a different origin. This case can be seen in Figure 9 (https://biocyc.org/META/NEW-IMAGE?type=PATHWAY&object=PWY-5913&detail-level=1) (accessed on 1 March 2020), where the destiny node succinate can only be reached by starting at the origin node phosphoenolpyruvate. Therefore, for this test to consider a pair of pathways as a candidate for a given comparison, there must be an actual valid traversal lecture between the same given origin and destiny nodes. It is worth mentioning that this also implies there can be more than one valid traversal or lecture for a given pair of pathways, as long as they meet both selection criteria.

A third argument applied to further filter the candidates for the experimental comparisons was to select only the data with *full-coverage traversals*. This refers to considering those graphs in which all nodes are covered in a single traversal or lecture, and we call this a fair comparison.

### 4.5. Graphs vs. Pathways

The characteristics of the graphs associated with each pathway and the pairwise comparison are taken into account as factors that could influence the results. These factors are also annotated for each comparison:

- *Size ratio between graphs*: for a given graph, we refer to the “size” as the number of nodes described within the graph. The size ratio between graphs consists of dividing the smaller graph’s size (graph with the least amount of nodes) over the bigger graph’s size (graph with the largest number of nodes) to obtain a value between 0 and 1 that represents how different the sizes of the compared graphs are. This value can also be interpreted as how much of the biggest graph’s size can be covered by the smallest graph’s size.
- *Complexity ratio between graphs*: is the number of edges described within a given graph. The complexity ratio between the graphs consists of dividing the complexity number of the less complex graph (graph with the least amount of edges) over the complexity of the more complex graph (graph with the largest number of edges) to obtain a value between 0 and 1 that represents how different the complexity amounts of the compared graphs are. This value can also be interpreted as how much of the more complex graph can be covered by the less complex graph.
- *Equivalent nodes ratio*: this is obtained by dividing the number of equivalent nodes over the size of the larger graph. It produces a value between 0 and 1 that can be interpreted as how much percentage of the equivalent-nodes present in the larger graph can also be found within the smaller graphs. Equivalent nodes mean that the same metabolite is present in both graphs.

### 4.6. Formulas

For the evaluation process in the next section, we define some formulas as the basis for our metrics of analysis.

- *Absolute Score*: S=xm+yn+zg, where *S* is the score, *x* is the number of matches, *m* is the value of a match, *y* is the number of mismatches, *n* is the value of a mismatch, *z* is the number of gaps, *g* is the value of a gap;
- *Relative Global*: $r_{G}$$=x_{G}$/max(|S|,|T|), where $r_{G}$ is the relative global score, $x_{G}$ is the number of matches of the respective global alignment, and *S* and *T* are the aligned sequences;
- *Relative Local*: $r_{L}$$=x_{L}$/min(|S|,|T|), where $r_{L}$ is the relative local score, $x_{L}$ is the number of matches of the respective local alignment, and *S* and *T* are the aligned sequences;
- *Relative Semiglobal*: $r_{Sg}$$=x_{Sg}$/min(|S|,|T|), where $r_{Sg}$ is the relative semi-global score, $x_{Sg}$ is the number of matches of the respective semi-global alignment, and *S* and *T* are the aligned sequences.

### 4.7. Executing Pairwise Comparisons

The pairwise comparison is generally any process of comparing entities in pairs to judge which of each entity is preferred or has a greater amount of some quantitative property, or whether or not the two entities are identical. In the psychology literature, for example, it is often referred to as a paired comparison.

Both graphs represent a different pathway traversed in a breadth-first manner for each pairwise comparison, starting at the selected “origin" node (as the root of the tree data structure) and concluding when all accessible leaves are reached, wherein a destination node can be found. As noted before, some nodes may get excluded from a particular traversal if they are located in a path only accessible by traversing from another “origin" node.

Each traversal lecture yields a linear sequence of the traversed nodes in the order in which they were visited. The first node of the sequence will always be the chosen origin, whereas the destiny node can be found anywhere in the sequence. The sequences obtained from the traversals are later aligned with global (GA) [4], local (LA) [25], and semi-global (SGA) sequence alignment algorithms. On the other hand, the Difference by Pairs algorithm is performed directly on the graph structures.

Then, we define different metrics for each pairwise comparison executed:

- *Global Score*: score generated by the absolute score formula for the optimal global sequence alignment between two traversals (Algorithm 1). The base values used were: match = 1 and mismatch = −1; for gap value, the comparisons were performed using 3 different values: −2, −1 and 0 (explained why later);
- *Relative Global Score*: value between 0 and 1 obtained from the relative global formula, a percentage value p%, interpreted as “there is a p% similarity between both traversals” or “at least p% elements of the small traversal is present in analogous order on the long traversal”;
- *Local Score*: score generated by the absolute score formula for the optimal local alignment between two traversals (Algorithm 1). The base values used were: match = 1, mismatch = −1, ga*p* = −2;
- *Relative Local Score*: value between 0 and 1 obtained from the relative local formula, a percentage value p%, interpreted as “at least p% elements of the small traversal is present in analogous order on the long traversal”;
- *Semiglobal Score*: Score generated by the absolute score formula for the optimal semi-global alignment between two traversals (Algorithm 1). The base values used were: match = 1, mismatch = −1, ga*p* = −2;
- *Relative Semiglobal Score*: Value between 0 and 1 obtained from the relative semi-global formula, a percentage value p%, interpreted as “at least p% elements of the small traversal is present in analogous order on the long traversal”;
- *Differentiation by Pairs*: For each one of the two pathways, a list of distinguished reactions present on the said pathway but absent in the other one was obtained as a result. Each reaction, or edge in the graph, is represented as a string of the form “node_0 -> node_1 ”, meaning a metabolite0 is being transformed into metabolite1;
- *Numerical Differentiation by Pairs*: For a given pairwise comparison between two pathways, consists of the total number of elements of the previous differentiation by pairs results, divided by the sum of complexity of both graphs. This provides a value between 0 and 1 that represents a percentage of the distinguished reactions (edges) constituted between the two graphs that are unique in each graph. The complexity of the graph should be understood as the number of reactions in a single pathway.

**Figure 10:**
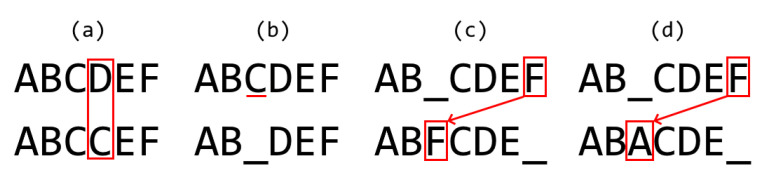
Relative global scores for different scenarios. (**a**) match, (**b**) gap, (**c**) double gap, (**d**) double gap and missmatch.

It is also essential to consider the further interpretation of relative global scores. Let us consider Figure 10:

All cases shown in Figure 10 have five matches over a maximum sequence length of 6, so all would be 83% (5/6) similar according to the Relative Global: $r_{G}$$=x_{G}$/max(|S|,|T|), formula. Therefore, a difference under this standard could mean a substitution (case a), a deletion or addition (case b), a transposition (case c), or deletion and addition (case d).

## 5. Results and Discussion

From the population of all available pathways (3241) on the database matching the previously defined criteria: origin and destiny defined, origin to destiny valid traversal, and full-coverage traversals; pathways were randomly selected for different analyses and the runs of the experiments.

### 5.1. Former Tests

We see that each comparison “match” comprises two distinct corresponding graphs with an origin node selected and a destiny node expected. Remember that each pathway may have different traversals from the same pathway, producing different comparisons for a single pathway. We considered pathways with a valid traversal between an origin (root node) and destiny (leave node) for both graphs for primary analysis purposes. For fair comparison criteria, we also considered full-coverage traversals only. It means that we are considering pathways as connected graphs.

For primary analysis purposes, we considered pathways with valid traversal between an origin (root node) and destiny (leave node) for both graphs. For fair comparison criteria, we also considered full-coverage traversals only. It means that we are considering pathways as connected graphs.

Taking into account all the data could mean millions of comparisons and many hours of computer work; to simplify the process, we considered a random statistical sample with selection criteria, and in the same proportion of elements presented in the total population data, they were: number of nodes (size) and one origin node (root) only (the latter guarantees full-coverage traversals).

**Figure 11:**
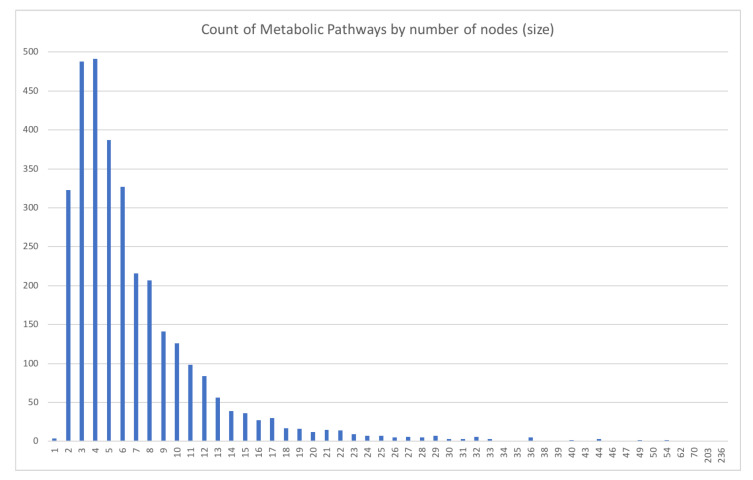
Quantity of Metabolic Pathways ordered by size.

**Figure 12:**
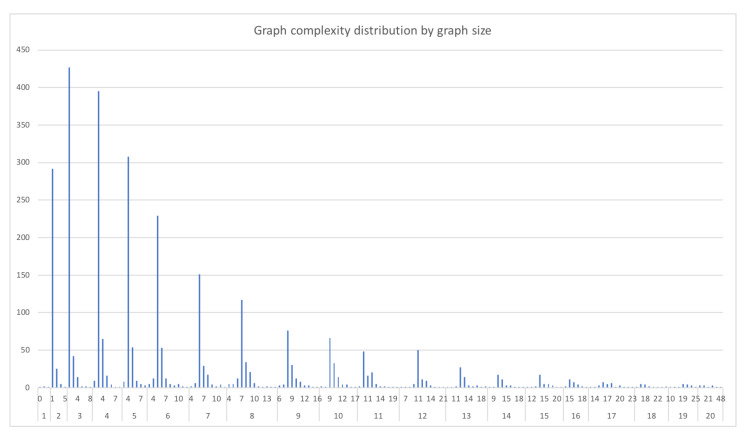
Metabolic Pathways ordered by size and its associated complexities.

In Figure 11 and Figure 12, we can see the data distribution categorized by graph size and complexity (edges), representing the number of pathways with each characteristic.

**Figure 13:**
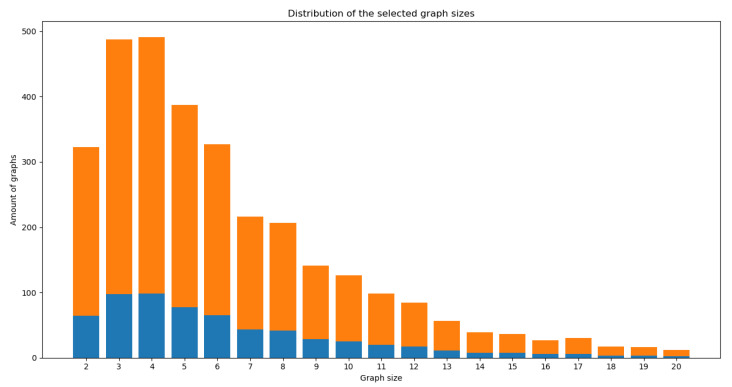
Size distribution quantity of metabolic pathways selected. The height of each bar represents the total pathways selected using the defined criteria of size and origin. Blue represents a statistic random sample of 20% of the dataset in a scale proportion of sizes.

For the first overview analysis, the most representative values selected were: size from 2 to 20 for 3125 pathways (96.4%); selecting the pathways with one origin only provides a total of 2340 pathways, distributed as shown by the bar’s height in Figure 13.

Then, we selected a random statistic sample of 20% (468 pathways) in a scaled proportion of the selected size criteria (2 to 20), meaning 20% of each of these sizes, as shown in Figure 13 in blue color.

The first run of 109,278 pairwise comparisons was executed. All pairwise comparisons were measured using the proposed algorithms. Also, each matching pair was tested using a third-party external tool. We reviewed many previous works to evaluate our results. Considering the similarity in the outputs, not all were available, updated, open-source, accessible, and so forth. We selected a tool that was available and also provided a pairwise comparison with a 1 to 1 score on a scale of 0.0 to 1.0. This tool is called “TMPAlign”, a newer version of the tool MPAlign introduced in 2014 [30].

**Figure 14:**
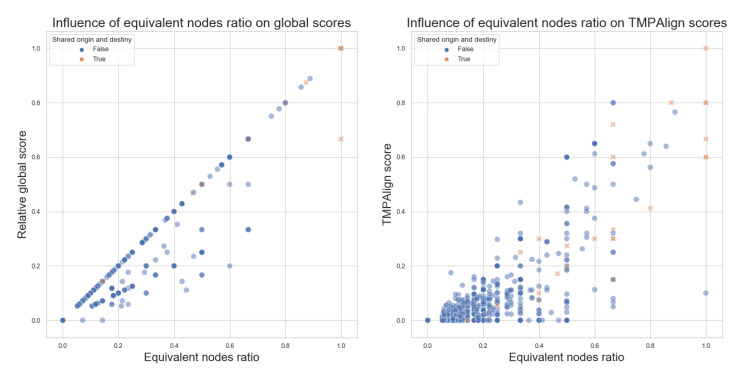
Equivalent nodes ratio vs. Relative Global Scores and TMPAlign scores, with a random statistic sample of 20%. Pairwise comparisons, where origin and destiny are corresponding equivalent nodes, are denoted with an orange X marker.

With the random statistical sample of 20% of the selected data, we can see in Figure 14 an exciting observation, as a first result: all of the pairwise comparisons reporting 0% equivalent nodes generate a score of 0 for all scores, for our Global Scores and even for the TMPAlign tool. So, for the rest of the comparisons, we are avoiding these comparisons where the Equivalent nodes ratio is 0 since they are not providing significant values and consume a significant amount of computation time in our batch runs that will finally score 0. This allowed a bigger statistical sample for subsequent runs, with more significant results.

### 5.2. Analysis of Algorithms for Pairwise Comparisons

Several execution metrics were conducted to evaluate the tests for each pairwise comparison previously defined. After the first tests with the sample of 20%, and considering that the comparisons without equivalent nodes always generate identical scores (0, as described before), the random statistical sample was increased to 50% while simultaneously only performing the comparisons with at least one equivalent node, so a broader diversity of metabolic pathways were tested. This new selection represented 1169 pathways, which means 682,696 possible comparisons.

The first algorithm mainly relies on the score provided by the global alignment; however, to provide a better meaning to this, some additional metrics were developed in order to adjust the relationship between the scores and the data, like the coverage of one pathway with others, especially when they are of different sizes. So, the values are indicated in a ratio relationship from 0.0 to 1.0.

The global score seeks to analyze the traversal or complete lecture of the pathway and consider the number of similar elements as a whole. In the tests carried out, it was observed that applying a negative gap assessment such as the −2 standard does not generate any meaning in a metabolic process as such, since a sequence obtained from a metabolic pathway does not lose any information during its lecture, as happens in DNA or RNA sequencing. Hence, the best values obtained, reflecting a more realistic evaluation as a pairwise comparison, was by using a gap value of 0 in the global score. Then, 0 was the gap value selected for the pairwise comparison for algorithm 1.

**Figure 15:**
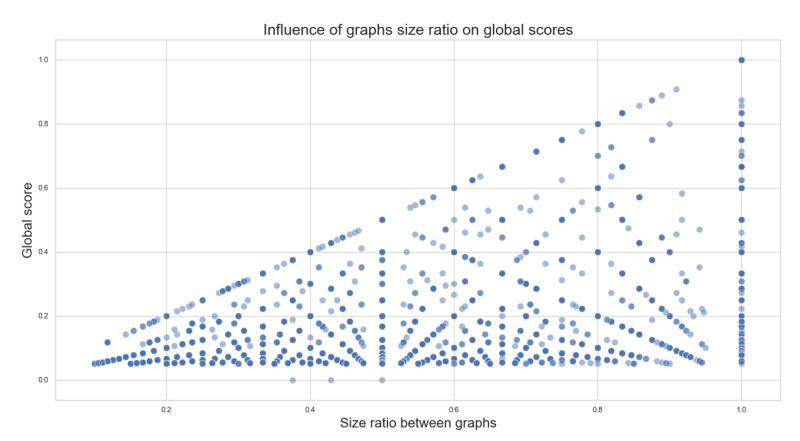
Influence of the graph size in the global comparison scores.

For the first algorithm, Figure 15 shows the relationship between the graphs’ size and relative Global pairwise comparison scores. We can see no direct correlation between the size of the graphs and the Global scores. It was found that this “disordered” behavior is expected in all algorithms tested, meaning independence between the data and the algorithms.

It is worth noting that, for the relative global score, the “highest possible score” is delimited to the size ratio of the graphs. For example, let us consider two different pathways, one with three nodes and one with ten nodes. The best chance of a good comparison here is that the nodes of the minor pathway are all in the bigger one, in the same order; the highest possible score, in this case, would be 30%. This can also be seen in Figure 15 as a “diagonal” that bounds the dispersion of the points across the graph.

**Figure 16:**
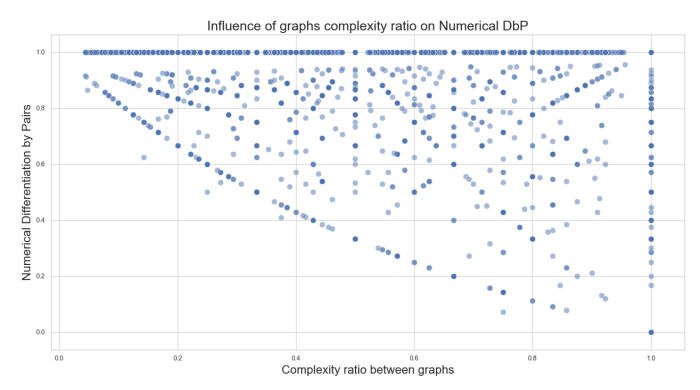
Influence of the graph complexity in the DbP pairwise comparison scores.

Then, for the second algorithm, Figure 16 shows the relation between the complex ratio of the graphs and its influence on Numerical DbP scores. The numerical evaluation of the second algorithm seeks the differences between the graphs, according to the difference in edges. We can see that there is no direct correlation between the complexity of the graphs and the numerical DbP scores either.

**Figure 17:**
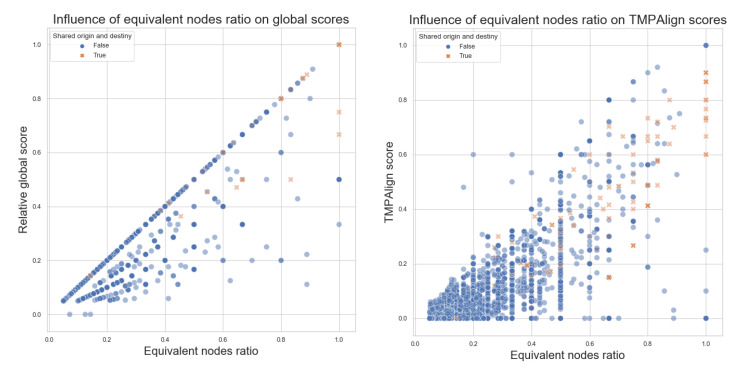
Equivalent nodes ratio vs. Global scores and TMPAlign scores, with a random statistic sample of 50% and excluding comparisons without equivalent nodes. Pairwise comparisons, where origin and destiny are equivalent, are denoted with an orange X marker.

Next, in Figure 17, we can see the relationship between the number of equivalent nodes and the Global scores of Algorithm 1 on the left side and for the TMPAlign tool on the right side, with the sample of 50%. Similar to the threshold observed for the global scores according to the size ratio in Figure 14, similar behavior can be seen with the equivalent nodes ratio, with more points being aligned in the central diagonal than before. This also occurs at a lesser degree for the comparison values obtained with TMPAlign, where we can observe that the scores are less correlated to the equivalent nodes ratio; this implies that TMPAlign could be considering other factors when generating its scores. Nevertheless, there is an important observation here: when the graphs should not be similar (i.e., at low equivalent nodes ratio), both tools tend to probe this.

**Figure 18:**
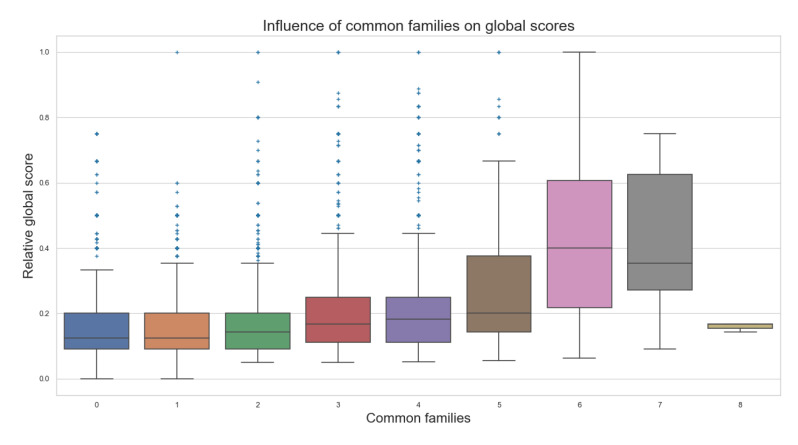
Common families on Global scores.

If we also consider the common families of the pathway, from a range of about 638 different families in the total population of 3241 pathways, it is easy to denote a great diversity of metabolic pathways. If we consider grouping the pathways in a common families criteria, we can observe in Figure 18 that most of the scores related to each category remain very close. As the compared pathways have more families in common, the scores tend to be higher. As all in biology, there is an exception to this. Some pairwise compared metabolic pathways with several common families, eight for the case shown in the figure, may have a few or no similar elements at all for comparison, producing lower scores than its counterparts, contradicting some of the general behaviors observed for most of the comparisons.

### 5.3. Experiments

As indicated, thousands of pairwise comparisons have been executed for different tests and analyses from different points of view. To have a more formal test using statistical analysis, some extra runs were conducted. Beyond the biological relevance of the data obtained from the algorithms, now we want to measure if there are some key elements from the input data that can impact or influence the score obtained in a result for a better understanding of some different characteristics of the proposed algorithms.

$$\begin{array}{ccc} Runs & \; & =\mathrm{Size}\;\mathrm{ratio}*\mathrm{Common}\;\mathrm{families}*\mathrm{Replicas}\\ \; & \; & =3*3*50\\ \; & \; & =450 \end{array}$$

Using the DoE methodology of [27], 450 runs of the experiment were completed. This number results from the multiplication of the factors described in Table 1 and 50 replicas for each combination. That is, 50 random comparisons of each possible crossover were executed.

All comparisons made were unique (i.e., no repetitions in the analyzed data), and all of them were randomly selected.

### 5.4. ANOVA Tests

A two-way ANOVA was conducted to examine the effects of size ratio and families on the relative global score of pairwise comparison of metabolic pathways.

Assumptions The ANOVA test makes the following assumptions about the data:

- Independence of the observations. Each subject should belong to only one group. There is no relationship between the observations in each group. Having repeated measures for the same participants is not allowed;
- No significant outliers in any cell of the design;
- Normality. the data for each design cell should be approximately normally distributed;
- Homogeneity of variances. The variance of the outcome variable should be equal in every cell of the design.

Residual analysis was performed to test for the assumptions of the two-way ANOVA. Outliers were assessed by the box plot method, normality was assessed using Shapiro–Wilk’s normality test, and homogeneity of variances was assessed by Levene’s test (Listing 1).

**Listing 1:** Two-way ANOVA: Global. Summary statistics. R output. (Own Source).

>
#* Summary statistics*
#* Compute the mean and the SD (standard deviation)*
#* of the Global score by groups:*
> doe_selection %>%
+ group_by(size_ratio, families) %>%
+ get_summary_stats(global, type = "mean_sd")
#* A tibble: 9 x 6*
size_ratio families variable n mean sd
<chr> <fct> <chr> <dbl> <dbl> <dbl>
1 different none global 50 0.095 0.043
2 different few global 50 0.101 0.048
3 different several global 50 0.135 0.07
4 medium none global 50 0.166 0.081
5 medium few global 50 0.166 0.094
6 medium several global 50 0.239 0.155
7 similar none global 50 0.199 0.131
8 similar few global 50 0.185 0.107
9 similar several global 50 0.321 0.161
>

**Figure 19:**
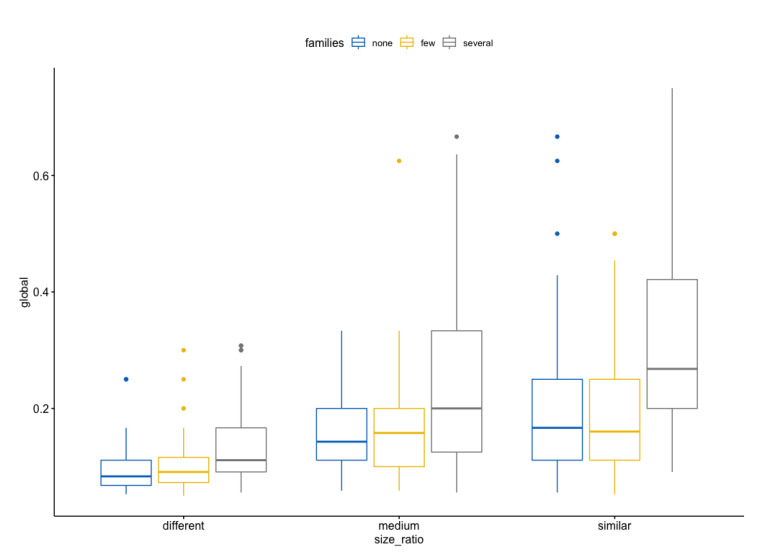
Global score by size ratio levels, colored by families levels.

Figure 19 summarizes this output.

There were some extreme outliers, residuals were not normally distributed (*p* > 0.05), and there was no homogeneity of variances (*p* > 0.05).

A data transformation using Log 10 was applied, shown in the following Listing 2. After that, there were no extreme outliers; residuals were normally distributed (*p* > 0.05). However, there was no homogeneity of variances (*p* > 0.05) for all the cases.

**Listing 2:** Log10: Transforming the Data. (Own Source).

>
#* Some common heuristics transformations for non-normal data*
# *include: log for greater skew:*
#* log10(x) for positively skewed data,*
#* log10(max(x+1) - x) for negatively skewed data*

# *Log transformation of the skewed data:*
> doe_selection**$**global **<- log10** (doe_selection**$**global)
>

**Figure 20:**
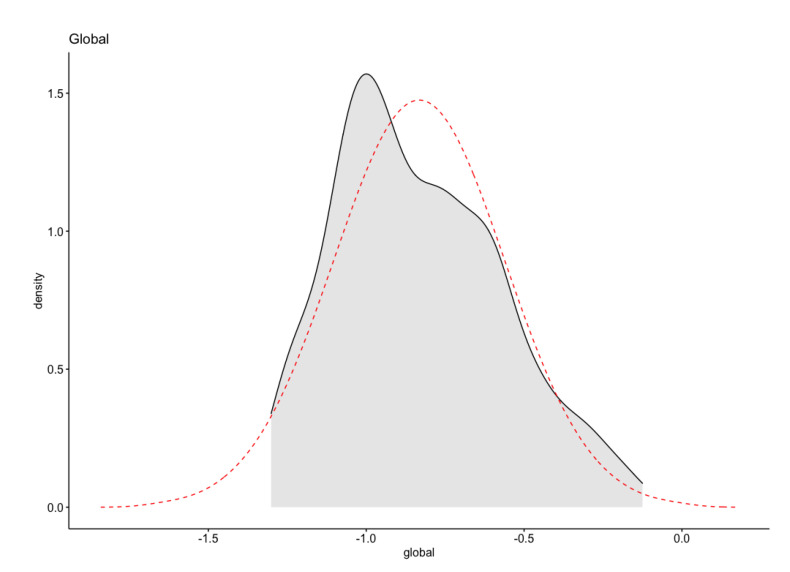
Normality test of Global Score: normal, after log10 transformation.

The log10 transformation improved the distribution of the data to normality as shown in Figure 20 (Listing 3).

**Listing 3:** Homogeneity of variance assumption test. (Own Source).

>
#* This can be checked using the test of Levene:*
> doe_selection %>% levene_test (global ~ size_ratio∗families)
#* A tibble: 1 x 4*
df1 df2 statistic p
<int> <int> <dbl> <dbl>
1 8 441 3.48 0.000657
>

Levene’s test is significant (*p* > 0.05), as shown above. Therefore, we cannot assume the homogeneity of variances in the different groups [28,31]. In all cases, you may want Levene’s Test statistic to be non-significant. In the case that it is significant, you can either:

(a) ignore this violation, based on your own a priori knowledge of the distributional characteristics of the population being sampled;
(b) relax the assumption of homoscedasticity and run the Welch one-way test, which does not require that assumption [32].

In this case, Levene’s test is testing whether the variances of the groups are significantly different. If Levene’s test is significant (i.e., the value of significance is less than 0.05), then we can conclude that the variances are significantly different.

If the overall *p*-value from the ANOVA table is less than some significance level, then we have sufficient evidence to say that at least one of the means of the groups is different from the others. However, this does not tell us which groups are different from each other. It simply tells us that not all of the group means are equal.

In order to find out exactly which groups are different from each other, we must conduct pairwise *t*-tests between each group while controlling for the family-wise error rate. One of the most common ways is to use Bonferroni’s correction when calculating the *p*-values for each pairwise *t*-tests [33].

The Welch test is an alternative to the standard ANOVA where the homogeneity of variance cannot be assumed (i.e., the Levene test is significant). In this case, the Games–Howell post hoc test or pairwise *t*-tests (with no assumption of equal variances) can be used to compare all possible combinations of group differences [28,31,32].

On the other hand, if the normality assumption is not met, we could consider running the statistical tests (t-test or ANOVA) on the transformed and non-transformed data to see any meaningful differences. If both tests lead you to the same conclusions, you might not choose to transform the outcome variable and carry on with the test outputs on the original data [34].

Tests were executed using transformed and non-transformed data; results were similar, showing evidence of interference of size ratio and families into the Global score.

The biological reference suggests that there will be higher scores when the compared data are more similar (size ratio = medium or similar) and there are some common families (some and several), as observed in the tests executed (Listing 4).

**Listing 4:** ANOVA Table after Bonferroni correction. (Own Source).

>
#* In the R code below, the asterisk represents the interaction*
#* effect and the main effect of each variable*
#* (and all lower-order interactions).*
> res.**aov** <- doe_selection %>%
+ **anova**_test(global ~ size_ratio ∗ families)
Coefficient covariances computed by hccm()
> res.**aov**
ANOVA Table (type II tests)
Effect DFn DFd F p p<.05 ges
1 size_ratio 2 441 52.229 4.39e-21 ∗ 0.192
2 families 2 441 27.777 4.35e-12 ∗ 0.112
3 size_ratio:families 4 441 3.209 1.30e-02 ∗ 0.028
>

Then, some extra tests were conducted: Welch One way ANOVA test, pairwise comparisons using Games-Howell, and pairwise comparisons using the pairwise t-test with no assumption of equal variances with Bonferroni correction [33]. They all show a significant interaction between the size ratio and families for the most representative combinations. When there are few or several common families, the size ratio is medium or similar.

**Figure 21:**
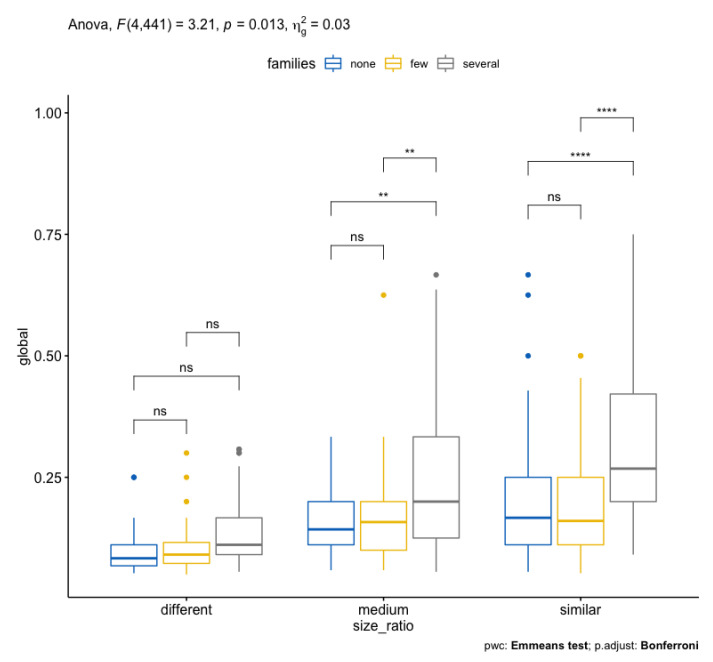
ANOVA summary: statistically significant interaction between size ratio and common families on Global score, F(4, 441) = 3.21, *p* = 0.013, eta2[g] = 0.03. Box plots with *p*-values. ns: not significant, **, ****: interaction levels (own preparation).

There is a statistically significant interaction between size ratio and common families on Global score, F(4, 441) = 3.21, *p* = 0.013, eta2[g] = 0.03. See the ANOVA table above (Listing 4) after applying a Bonferroni adjustment [33]. The ANOVA Summary is shown in Figure 21.

Consequently, an analysis of simple main effects for size ratio was performed with statistical significance receiving a Bonferroni adjustment. There is a statistically significant difference in mean “Global” scores for both medium (F(2, 441) = 7,72, *p* < 0.05) and similar (F(2, 441) = 24.4, *p*< 0.05) size ratio to either none–several or few–several common families levels.

As can be expected from the biological point of view, when there are no common families between the compared metabolic pathways, the scores are not influenced and are statistically demonstrated. It is the same situation when the size ratio between pathways differs for any family category.

All pairwise comparisons were analyzed between the different families’ groups organized by size ratio with Bonferroni correction. There was a significant difference in Global scores between the relation of groups for none–several and few–several relations (*p* < 0.05).

### 5.5. Tests against Another Algorithm of Reference

The TMPAlign algorithm that was selected as a tool for comparison was outdated. It was made available in 2017, written in a python version 2.7 using services of the KEGG database that are not available today as expected. Documentation of the tool points out it can work with any database, so it was adjusted to work with the same data files from MetaCyc used in this work. TMPAlign was also not using the data about enzymes (i.e., when comparing two reactions, it only considers the reaction’s id when generating the score) because the service required from KEGG to handle this information is not currently available as a free service. The data obtained from MetaCyc does not fulfill the same criteria. Furthermore, it is worth noting that, for some pairwise comparisons, the tool TMPAlign raised errors during the execution, with no clear explanation. All these errors were excluded from the subsequent analysis for all algorithms.

It is important to remark that the main goal of using a reference tool is not to generate the same values but instead prove that, if two pathways are significantly different, both tools can denote it, and the contrary for similar pathways.

As shown in previous sections, the TMPAlign tool was used in all the executed pairwise comparisons made. The proposed algorithms and TMPAlign tools show similar and comparable behaviors. The main aspect to consider here is that when a pair of metabolic pathways are compared, we want a value of valid comparison. When pathways have a similar structure and similar inside elements, we expect to have higher values from that interception. When there are a few or non-common nodes at all, we expect to have lower values to zero. It can be observed from all shown graphs that Algorithms 1 and 2, compared to TMPAlign, have similar behaviors, and they are comparable. The next section will demonstrate that the proposed algorithms are up to 10 times faster than TMPAlign.

### 5.6. Timing Evaluations

**Figure 22:**
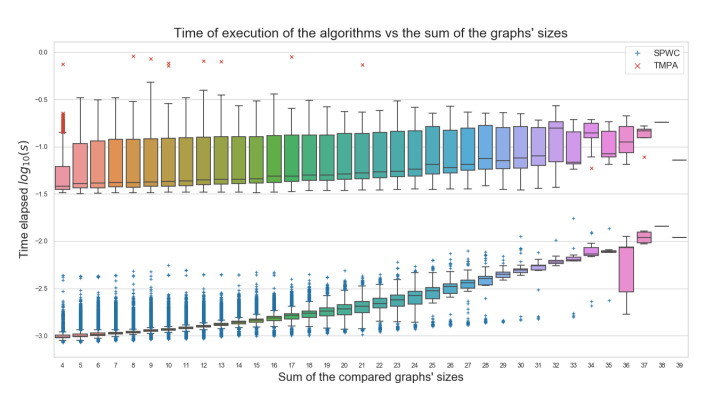
Execution running time comparison between tools and graph size. (Own preparation).

As regards the cost related to the execution time of the algorithms, this is one of the most important gains obtained with the proposed algorithms. Figure 22 shows the time consumption between our algorithms and the tool TMPAlign and its relation with the graph size. For this comparison, consider including the summarized timing of our algorithms and tested versions, all simultaneously, for each pairwise comparison and not a single execution at a time. So, we can see here the sum of the execution times of our algorithms versus a single run of TMPAlign. Even with the accumulated times, our algorithms show an improvement, being, on average, at least 10 times faster than TMPAlign. This was one of the most important goals defined for this work. As shown, the proposed algorithms are providing biologically significant information in a much faster way.

### 5.7. Summary

By way of summary, we propose the following points as results of interest:

- Data from a real, updated, and important database was used;
- The proposed algorithms execute at least 10 times faster than the external tool used for comparison. One of the main goals for this work;
- Equivalent nodes ratio equals 0, meaning no common elements between the pathways being compared, will always produce a 0 score;
- The more similar the sizes of the metabolic pathways and the more common families between them, the better the scores obtained;
- This is an observation that has biological meaning, with few exceptions;
- As with everything in Biology, there is no rule for 100% of the cases;
- The data does not follow a normal distribution;
- After a log10 transformation, a normality test shows positively skewed data;
- Homogeneity of variance was not perfectly adjusted;
- The analysis of variance was performed Welch’s ANOVA and Bonferroni correction to the models;
- Thousands of comparisons executed indicated independence in the input data and the resulting scores, but there is significant statistical evidence of the influence of the size ratio and common families;
- Data may have some extreme outliers. It was observed that pairwise compared metabolic pathways with several common families might have a few or none similar elements for comparison, producing lower scores than its counterparts, contradicting some of the general behaviors observed for most of the comparisons;
- Several scientific publications have been produced as a result of this work;
- The algorithms have been applied and extended to real scenarios with data from labs, confirming the biological significance of the proposed algorithms.

## 6. Conclusions

The pairwise comparison of metabolic pathways is an enormous yet interesting problem. In this work, we have attempted to provide some insight about it, implementing alternative methods to simplify how the data are used and, by doing so, sacrificing precision on the information representation but not on the results.

The comparisons that report 0% equivalent nodes unanimously report a 0% similarity under any of the evaluated algorithms, meaning that, when there are no equivalent nodes between the graphs, it can be safely reported that both pathways are completely different, without the need for executing the comparison algorithms.

It was shown that structural characteristics of the graphs, such as several nodes (size) or some edges (complexity), do not bias the comparison results when using the algorithms, as should be for a reliable tool. The only influence these factors have over the results is that, when comparing graphs with different sizes or complexities, the relative global score or numerical DbP score, respectively, will be penalized (decreased) because of the structural difference, whether the nodes or edges are similar or not. This is naturally to be expected, as these considerations are part of the design for the scoring system. In other words, the scores obtained from every pairwise comparison are dependent on the inner data nodes; two comparisons of graphs with equal size might produce different scores.

On the other hand, some characteristics obtained from the pairwise comparisons slightly affect the comparison results, such as the equivalent nodes ratio between the compared graphs, how many metabolic categories share the graphs, and whether the graphs have an equivalent origin and destiny nodes. When the first two have their numerical value increased, the comparison scores tend to increase as well; similarly, when the latter characteristic checks to be true, the comparison scores tend to be higher. It is also to be expected, as said characteristics hint at the pathways related. Likewise, it is important to denote that this tendency is not always the case. There can be comparisons that do not follow this pattern, which is also perfectly normal and shows how diverse the metabolic pathways and their comparisons can be.

The algorithms *Transformation of the 2D pathway graph to a 1D or linear structure for later alignment and evaluation* and *Algorithm 2: Differentiation by pairs* were also shown to be extremely resource-efficient, surpassing the speed of execution and in a more predictable manner than the tool of reference. It means that the goal of the algorithms (to simplify the graph comparison problem for it to be computationally lighter but still reliable) was achieved. The algorithm’s faster, more reliable, and more predictable behavior also mean that the tool can be successfully employed for batch comparisons, using large datasets of metabolic information, even though this was not the original intended use for the algorithms.

## 7. Future Work

Having verified that the proposed algorithms can provide relevant information for the analysis and comparison of metabolic pathways, it would be useful to implement a complete software tool capable of directly accessing metabolic databases, extracting information from metabolic routes of interest, and finally applying the proposed algorithms to the benefit of experts.

Other techniques for sequence comparison based on valuation matrices have been proposed so that not only the elements that are the same or different are evaluated using the same alignment values for all elements but rather that the affinity between elements is also considered. One example is seen in the case of protein sequence alignments, where if the amino acids are similar: hydrophobic, polar (positive or negative), and so forth, as well as if their energy and likelihood to react are similar, the substitution penalty will be lower than that when the nature of the amino acid is changed. It would be interesting to consider the comparison of pathways aspects such as those mentioned before and, therefore, provide even more information to the researcher.

Next, the interactions between routes should then be considered. Metabolites may be the final product of a route or an intermediate product which may be a precursor for other metabolic pathways. The analysis should be extended to combine these routes treated in their context as metabolic networks. As another level of comparison, enzymatic reactions that occur in each step of the metabolic process could be added into the pathways as extra information to be considered.

Finally, just as there are multiple alignment algorithms for several genetic sequences, it is important to continue working on the problem of comparing multiple pathways to find, for example, similar factors among different species.

## Figures and Tables

**Table 1:** Factors and levels analyzed.

	Factors	
	Size Ratio	Common Families
Levels	different	none
	medium	few
	similar	several

## Data Availability

Not applicable.

## Abbreviations

The following abbreviations are used in this manuscript:

BFT	Breadth-First Traversal
DbP	Differentiation by Pairs
DFT	Depth-First Traversal
